# Antimicrobial resistance: one world, one fight!

**DOI:** 10.1186/s13756-015-0091-2

**Published:** 2015-11-18

**Authors:** Stephan Harbarth, Hanan H. Balkhy, Herman Goossens, Vincent Jarlier, Jan Kluytmans, Ramanan Laxminarayan, Mirko Saam, Alex Van Belkum, Didier Pittet, for the World Healthcare-Associated Infections Resistance Forum participants

**Affiliations:** Infection Control Programme and WHO Collaborating Centre on Patient Safety, Geneva University Hospitals and Faculty of Medicine, Rue Gabrielle-Perret-Gentil 4, Geneva, Switzerland; King Saud bin Abdulaziz University for Health Sciences, College of Medicine, Riyadh, Saudi Arabia; Department of Medical Microbiology, Vaccine and Infectious Disease Institute, University of Antwerp, Wilrijk, Belgium; Laboratory of Bacteriology-Hygiene, Assistance Publique-Hôpitaux de Paris, Hôpital Pitié-Salpêtrière, Université Pierre et Marie Curie-Paris 6, Paris, France; University Medical Center Utrecht, Julius Center for Health Sciences and Primary Care, Utrecht, Netherlands; Center for Disease Dynamics, Economics & Policy (CDDEP), Washington, DC USA; Communication in Science, Rue des Maraîchers 8, Geneva, Switzerland; BioMérieux, Microbiology R&D Department, La Balme Les Grottes, France

**Keywords:** Antimicrobial resistance, Antimicrobial conservation, Antibiotic stewardship, One health, Infection control, Hand hygiene, Surveillance networks, Animal health, Global health, World healthcare-associated infections resistance forum

## Abstract

The lack of new antibiotic classes calls for a cautious use of existing agents. Yet, every 10 min, almost two tons of antibiotics are used around the world, all too often without any prescription or control. The use, overuse and misuse of antibiotics select for resistance in numerous species of bacteria which then renders antimicrobial treatment ineffective. Almost all countries face increased antimicrobial resistance (AMR), not only in humans but also in livestock and along the food chain. The spread of AMR is fueled by growing human and animal populations, uncontrolled contamination of fresh water supplies, and increases in international travel, migration and trade. In this context of global concern, 68 international experts attending the fifth edition of the World HAI Resistance Forum in June 2015 shared their successes and failures in the global fight against AMR. They underlined the need for a “One Health” approach requiring research, surveillance, and interventions across human, veterinary, agricultural and environmental sectors. This strategy involves concerted actions on several fronts. Improved education and increased public awareness are a well-understood priority. Surveillance systems monitoring infections need to be expanded to include antimicrobial use, as well as the emergence and spread of AMR within clinical and environmental samples. Adherence to practices to prevent and control the spread of infections is mandatory to reduce the requirement of antimicrobials in general care and agriculture. Antibiotics need to be banned as growth promoters for farm animals in countries where it has not yet been done. Antimicrobial stewardship programmes in animal husbandry have proved to be efficient for minimising AMR, without compromising productivity. Regarding the use of antibiotics in humans, new tools to provide highly specific diagnoses of pathogens can decrease diagnostic uncertainty and improve clinical management. Finally, infection prevention and control measures – some of them as simple as hand hygiene – are essential and should be extended beyond healthcare settings. Aside from regulatory actions, all people can assist in AMR reduction by limiting antibiotic use for minor illnesses. Together, we can all work to reduce the burden of AMR.

## Background

Few people outside the medical field know that life-saving interventions such as chemotherapy, organ transplants, major surgery, and treatment of auto-immune diseases or infections in newborns rely on effective antimicrobials [1]. Beyond humans, there are also billions of pets, livestock and fish that depend on these agents, whether as therapeutic or prophylactic agents, or as growth promoters of questionable value. Nevertheless, every time we use antibiotics, we create a selective pressure for bacteria to mutate or exchange pieces of DNA and possibly develop drug resistance.

Worryingly, global consumption of antibiotics soared by nearly 70 % between 2000 and 2010 [2]. In developed countries, between 10 and 20 courses of therapy are prescribed to each individual before the age of 18 [3]. Every 10 min, almost two tons of antibiotics are used around the world, all too often without any prescription or control [4]. Overuse and misuse of these agents increase AMR; as a result, every 10 min a patient dies in the USA or Europe because antibiotics no longer were effective against the bacteria responsible for the infection [5, 6]. Such figures are unknown in other jurisdictions, but they are likely to be substantially higher in African and Asian countries.

The burden of AMR at the global level is still poorly quantified. As yet, no national death register records “deaths caused by antimicrobial-resistant infection”, with the notable exception of England & Wales [7]; therefore, officially no one has ever died of it. AMR places both humans and animals alike at greater risk for prolonged disease or death from bacterial infection [8]. Part of this increased mortality results from the more complex and less effective antimicrobial treatments needed for multidrug-resistant infections. Virulence factor genes that ride with AMR genes on mobile genetic elements might contribute to the problem [9].

### The recent momentum in policy initiatives to fight antimicrobial resistance

Time is of the essence for tackling AMR: the longer action is delayed, the harder control is to achieve in the long run [10]. As early as the 1990s, a small number of countries deployed national strategies and action plans to mitigate this threat, and some of these successfully reduced antibiotic consumption in humans and animals, as well as local rates of AMR. But most countries confronted this problem only more recently.

In 2013, the Global Risks Report of the Word Economic Forum stated: “one of the most effective and common means to protect human life – the use of antimicrobials – may no longer be readily available in the near future” [11]. That same year, science ministers attending the G8 Summit identified AMR as the “major health security challenge of the 21st century” [12].

In 2014, India banned over-the-counter sales of antibiotics in March, and in April, the World Health Organization (WHO) published its first global report on AMR [13]; in July, the British Prime Minister David Cameron commissioned a prominent economist – Jim O’Neill – to lead a review on the topic [14]. In September 2014, the United States announced a 5 year plan to combat the problem domestically and internationally; 6 months later, the Obama administration committed a historic investment to reduce inappropriate antibiotic use by 50 % in outpatient settings and 20 % in inpatient settings by 2020, using 2011 as a reference [15]. Furthermore, the USA have established a Presidential Advisory Council on Combatting Antimicrobial Resistant Bacteria, that is charged with monitoring and coordinating the actions of the various federal agencies engaged in tackling AMR.

The 2015 G7 summit called again for intensive international collaboration in this field, endorsing WHO's global action plan and One Health approach [16]; but it remains to be seen if and how this call will translate into financial resources and concrete actions [17]. Finally, in a situational analysis published in April 2015 [18], WHO determined the extent to which effective practices and structures to address AMR have been set up across the world and where gaps remain. This country survey focused on the prerequisites to combat AMR and revealed that the task ahead is daunting. However, the emerging global trend is encouraging and there are success stories – and best practices – to be shared.

This momentum brought together 68 world experts at the fifth biennial World Healthcare-Associated Infections Forum in Annecy, France, on 14–16 June 2015. Experts gathered to address AMR control in low, middle, and high-income countries using a One Health perspective. This article summarises the contributions presented during that Forum following the strategic objectives of WHO’s Global Action Plan on AMR (Table 1) [19].

**Table 1:** Strategic objectives of WHO’s Global Action Plan on Antimicrobial Resistance

Objectives	Means
Improve awareness and understanding of AMR	Communication, education and training
Strengthen the knowledge and evidence base on AMR	Surveillance and research
Reduce the incidence of infections	Sanitation, hygiene and infection prevention measures
Limit the emergence and spread of AMR	Optimal use of antimicrobial medicines in human and animal health
Develop new tools to fight AMR	New medicines, diagnostic tools, vaccines and other interventions.

## Communication, education and training

Evidence for the effectiveness of public campaigns targeting antibiotic use is still weak, as in most cases their follow-up evaluations have been limited [20]. Although awareness campaigns do not provide miracle solutions, some of them have been quite successful.

Following Belgium’s national awareness campaigns, resistance of *Streptococcus pneumoniae* to penicillin decreased from 18 % in 2000 to 7 % in 2009. The total number of antibiotic packages consumed per 1000 inhabitants decreased from 3.6 in 1999–2000 to 2.4 in 2009–2010 (−33 %). Between 2002–03 and 2008–09, social security expenditures for the reimbursement of these drugs dropped by EUR 21 million (−16.7 %). As total expenditure for the six national campaigns between 2002 and 2009 amounted to EUR﻿ 2.4 million and cumulative savings through this same period reached EUR﻿ 90 million, for every euro invested in the campaigns around EUR 25 were saved [21]. With its new Strategic Plan 2014–2018 aiming at a 5 % annual reduction in antibiotic packages consumed, Belgium anticipates potential cost savings amounting to EUR 35 million by 2020.

France’s national campaign between 2002 and 2007 decreased the total number of antibiotic prescriptions per 1000 inhabitants by 26.5 %, with the greatest reductions (−35.8 %) recorded among children aged 6 to 15 years. Measured in Defined Daily Doses (DDD) per 1000 inhabitants, the corresponding drop in outpatient antibiotic consumption was less significant, from 32 to 28.5 DDD per 1000 inhabitants per year over the period [22]. The country still registers high consumption rates relative to its neighbours, and the impact of the 2002–2007 campaign is fading, with consumption picking up again especially among the elderly [23, 24].

In 2007, the European Union pioneered an annual “Antibiotic Awareness Week” and “Antibiotic Awareness Day”, which was accompanied by a similar effort, “Get Smart Week” in the USA in 2008. Canada also joined in 2010, followed by Australia in 2012 and New Zealand in 2014. Surprisingly, outpatient antibiotic prescriptions in Europe seem to have peaked in 1997, well before the implementation of national campaigns on the subject. Nevertheless, public campaigns have had an impact on the rate of antibiotic-related consultations and prescriptions [24], with an average reduction of consumption of 1.3–5.6 daily doses per 1000 inhabitants [25]. In the future, the EU Commission ambitions to target media campaigns more effectively at those who lack knowledge, and at prescribers and pharmacists who have a key role to play in changing views and behaviour [26].

### Assessing the effect and determinants of success of public awareness campaigns

Monitoring the impact of awareness or educational campaigns remains challenging. The choice of indicators to monitor campaign outcomes is crucial and for some comparisons the number of antibiotic packages sold is more appropriate than the commonly used number of DDD per 1000 inhabitants [27]. Showing potential savings to decision-makers can be a strong catalyst for decisions and funding, although economic indicators can be unhelpful because some broad spectrum antibiotics (e.g. amoxicillin/clavulanic acid) may be cheaper than narrow spectrum antibiotics (e.g. flucloxacillin), and prices may be driven up or down by rapidly evolving production capacities.

Factors leading to successful awareness campaigns include carefully designed and simple key messages; targeting patients, their families and healthcare workers; engaging physicians early in the campaign and designing the key messages with them; using mass media and social media; and continuously repeating key messages [20, 28].

Despite all the efforts being conducted at the scientific and regulatory levels, one important factor neither given significant attention nor investment is the human factor. Behaviour, whether through individualism, disconnect, or lack of proper education, is a significant obstacle moving forward. Increased investments are needed to not only survey the current behavioural landscape, but also to define strategies to enact effective change.

### Antimicrobial resistance and education

Reinforced education of medical students about the conservative prescription of antibiotics is crucial [13, 29]. But beyond undergraduate and graduate medical curriculums, training of pharmacists, nurses, midwifes and dentists, and of veterinarians, veterinary nurses and technicians, also needs to be reinforced. All these groups of professionals may be in a position to prescribe antibiotics – or influence prescriptions – in certain situations [25, 30].

The use of contemporary technologies for education and training proved highly valuable [31]. Massive Open Online Courses (MOOCs) can reach a wide array of professionals across the world, and smart phones provide an effective platform for antimicrobial stewardship applications [32, 33]. Instead of creating new programmes from scratch, existing good quality MOOCs or apps can be adapted to local needs.

As educational activities are rarely sufficient to change behaviour, additional cross-disciplinary research involving psychologists, medical anthropologists, sociologists and ethnographers is needed to find the most efficient means to modify behaviours and achieve “culture change”. This type of research needs to be supported at a global level and fine-tuned at the local level. It also requires leadership from ministers of health, leaders in healthcare settings, and professional societies.

## Surveillance and research

### Global antibiotic use and antimicrobial resistance

**Fig. 1:**
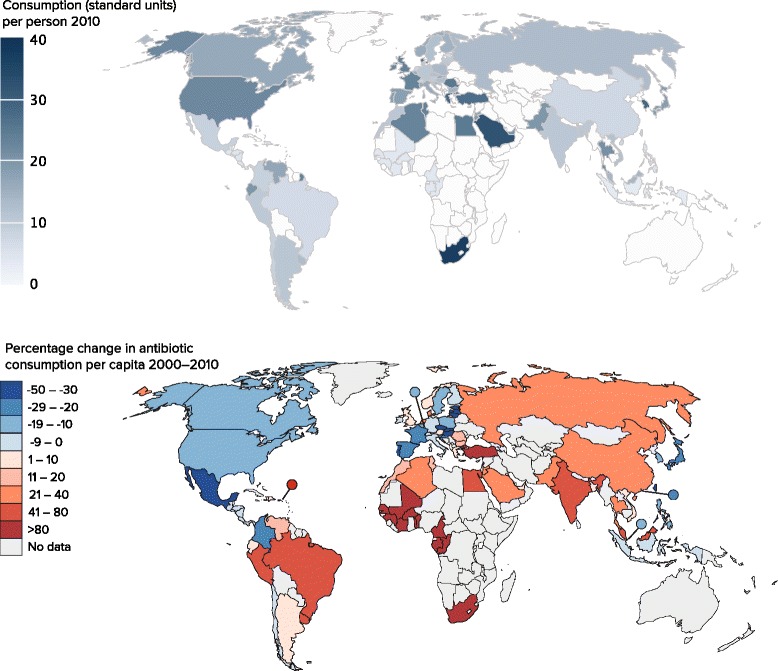
Consumption of antibiotics in 2010 and evolution of consumption per capita between 2000 and 2010. Legend: Consumption of antibiotics in 2010 expressed in standard units (ie, pill, capsule, or ampoule) per person, and percentage change in consumption per capita between 2000 and 2010. Reproduced with permission from CDDEP (Resistance Map: http://resistancemap.cddep.org/). Source: IMS MIDAS International Prescription Data, January, 2000–December, 2010, IMS Health Incorporated. All Rights Reserved. The statements, findings, conclusions, views, and opinions contained and expressed herein are not necessarily those of IMS Health Incorporated or any of its affiliated or subsidiary entities

The Center for Disease Dynamics, Economics & Policy (CDDEP) has surveyed human consumption of antibiotics, finding a 36 % per capita increase between 2000 and 2010 [2]. Whereas consumption decreased in countries such as Mexico or Chile, it sharply increased in many low-income and middle-income countries (LMIC). Figure 1 summarises the annual growth rate in antimicrobial use in 69 countries. Globally, colistin and carbapenem availability and consumption have increased massively [2]. At country level, the main drivers of increased antibiotic consumption are rising incomes (as more people demand antibiotics for minor infections), insufficient investment in public health capacity, and a significant background of infectious diseases [34].

Increasing worldwide demand for meat has led to antibiotic consumption in animals rising by 70 % over the past decade [35]. As a result, two thirds of antimicrobial consumption globally is linked to farm animals, often with limited oversight by trained veterinary personnel [36]. Mexico, Brazil, Australia, New Zealand and Vietnam have adopted and implemented partial bans on the use of antimicrobials for promoting growth in farm animals. The European Union banned such practices in 2006 [37], but by 2012 some 8000 tons of antibiotics were still being delivered to animals, with large variations between countries [38]. In the United States, recent policy changes in the veterinary field include a ban on the use of fluoroquinolones for poultry production and a ban on off-label administration of cephalosporins to food animals [39]. But for the other antibiotic classes used for growth promotion, withdrawal is voluntary and no limits have been imposed on their routine and often continuous in-feed or in-water use for disease prevention or therapy. On a voluntary basis, a growing number of food companies are banning the use of meat produced using antibiotics to promote growth, and the efficacy of these practices on weight gain is marginal and dwindling [40].

From March to June 2014, WHO performed a global Point Prevalence Survey of multidrug-resistant (MDR) organisms in healthcare settings. Through an online survey, it collected data from 420 laboratories in 67 countries on selected bacterial pathogens isolated from inpatient clinical blood and urine samples. Up to 10 % of the participating laboratories did not fulfill minimum criteria for quality controlled detection of AMR and thus were excluded from the analysis. This survey revealed wide regional variations for extended-spectrum β-lactamase (ESBL)-producing *Escherichia coli* and *Klebsiella spp* isolates (11.8–58.5 % and 35.1–57.3 %, respectively) and methicillin-resistant *Staphylococcus aureus* (MRSA) (27.7–44.4 %).

As an outcome of the 4th World HAI/Resistance Forum held in 2013, the Global Point Prevalence Survey (Global-PPS) was launched in 2014 to monitor rates of antimicrobial prescription and resistance in hospitals worldwide [41]. The Global-PPS is based on a standardised protocol and validation process to ensure the collection of comparable data among adults, children or neonates across different types of wards. A simple web-based interface allows data entry from a wide range of geographical settings, and returns detailed analyses of antibiotic use and prescription practices. It also provides benchmarking tools at national, continental and global levels, and identifies potential areas for improvement. All tools are freely available to any hospital in the world (www.global-pps.com). This survey invited hospitals worldwide admitting adults, children and neonates, to volunteer to participate. Data collected included age, gender, weight, antimicrobial agents, doses, reasons and indications for treatment, microbiological data, compliance to guidelines, documentation of reasons and stop/review date of prescription. Denominators included the total number of inpatients. A web-based application was used for data-entry, validation and reporting. Time frame of data collection was from February until September 2015. As of June 2015, 223 hospitals from 44 countries in Africa, Asia, Europe, North- and South-America and Oceania, participated. The final results will be released in November 2015 and are expected to include data from 700 hospitals in more than 70 countries.

### Antibiotic use at national level

Data from the European Surveillance of Antimicrobial Use and the European Antimicrobial Resistance Surveillance System have shown that European Union countries have been at least partially successful in controlling AMR. The various rotating European presidencies have allowed bottom-up initiatives to gain momentum within member states and to translate into Council recommendations. Strong leadership, with close links between opinion leaders, policy makers and politicians in support of AMR research has also provided evidence for successful public health interventions.

In Australia, data from the national surveillance system has revealed a rather high outpatient antibiotic use, at some 25 DDD per 1000 inhabitants per day, as compared to European countries (20 DDD per 1000 inhabitants per day). Conversely, the quinolone use in outpatients is among the lowest worldwide, at about 0.4 DDD per 1000 inhabitants per day [42].

A country-wide Point Prevalence Survey performed by the US Centers for Disease Control and Prevention (CDC) has estimated that around half of hospitalised patients receive antibiotics, and a quarter receive at least two classes of antibiotics. Only four drugs and a handful of conditions accounted for nearly half of the total antimicrobial use [43].

Countries of the Arabian Peninsula have reported a significant increase in antimicrobial resistance over the past decade [44–46]. Unrestricted dispensing of antimicrobial agents in both humans and animals are critical factors driving such resistance. In addition, the lack of knowledge of some physicians about antibiotics and their potential side effects continues to drive over-prescription [32].

### Antimicrobial resistance at national level

Data gaps are largest where health systems are weakest. As a result, the burden of drug-resistant infections in LMIC remains poorly described, but appears to be greater than in high-income countries [47].

At the global level, the worst threat comes from the emergence and rapid spread of MDR Gram-negative bacteria. It is a common concern in intensive-care units across Europe [48], and in Latin America and most of the Arabian Peninsula countries, in which Gram-negative bacteria have become more frequent than Gram-positive bacteria in hospital-acquired bloodstream infections, with a significant proportion of MDR strains [49, 50].

In particular, carbapenem-resistant Enterobacteriaceae (CRE) are of major concern, as few treatment options exist. In the Arabian Peninsula, the proportion of MDR Gram-negative bacteria exceeds 50 % in some hospitals, with carbapenem-resistant and ESBL-producing pathogens constantly rising [45]. Facing similar problems in 2010, the Greek island of Crete set up surveillance through the PROCRUSTES programme, which had considerable success in containing the incidence of MDR Gram-negative bacteria in intensive-care units. In Croatia, the national surveillance system in place since 1996 played a key role in containing an outbreak of carbapenem-resistant *Acinetobacter baumannii* in 2011. Unfortunately, many hospitals worldwide still lack the required resources for the adoption of effective infection prevention and control measures, such as isolation or cohorting capacities [51]. They also lack methods to ensure the proper initiation, justified continuation and prompt de-escalation of antibiotics use, which collectively are referred to as antimicrobial stewardship programmes.

The role of asymptomatic carriers of antibiotic resistance genes among humans and animals is of concern. A study on the prevalence, socio-demographic and hygiene profile of patients at a tertiary-care public hospital in Pakistan concluded that poor access to sanitation was an important predictor of carbapenemase carriage [52]. It revealed that 95 % of patients carried some form of AMR, and 20 % of these carried a New Delhi metallo-β-lactamase (NDM) variant of CRE. Fortunately, many of the bacteria carrying these forms of resistance were not clinically relevant, but their ubiquity is worrisome as a potential source of horizontal gene transfer to pathogens. The study did not find carbapenemase carriage being driven by previous antibiotic consumption, but among NDM carriers, previous exposure of carriers to heavy metals was a strong explanatory factor; mobile genetic elements carrying NDM often also carry genes conferring resistance to heavy metals [52].

Finally, CRE are also spreading among humans in the community with worrying implications for public health. The prevalence of carbapenemases in the healthy population in India has been estimated at 7.4 %, and its incidence in intensive-care unit patients at 27.4 % [53]. Other forms of resistance among Gram-negative bacteria not related to β-lactams or carbapenems are also becoming critical in India and in other countries, with resistance rates as high as 73 % against fluoroquinolones in *E. coli* [54].

### Spread of antimicrobial resistance between humans, animals and the environment

**Fig. 2:**
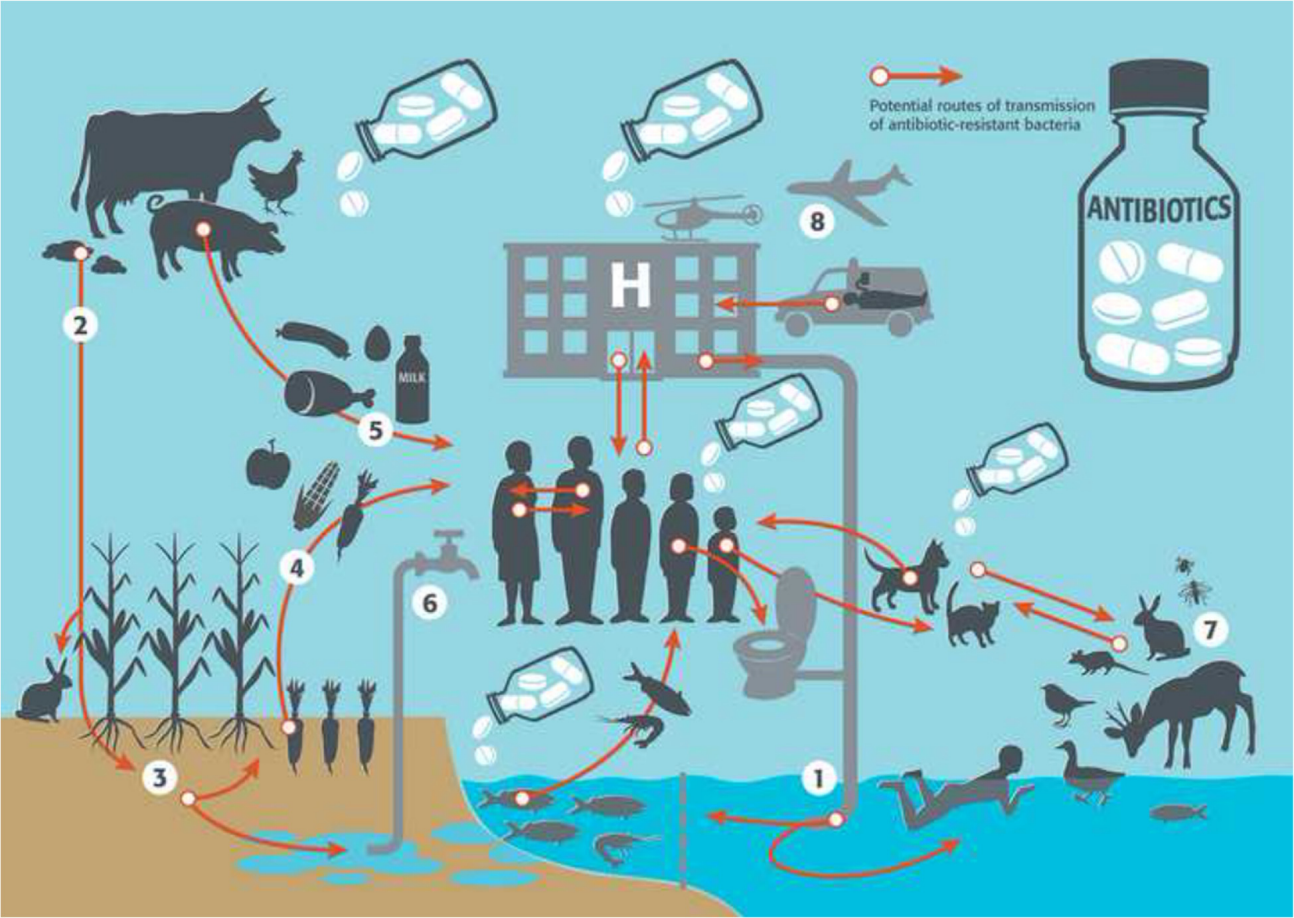
Potential routes of transmission of antibiotic-resistant bacteria. Legend: Humans in the community or in hospitals, pets, livestock and fish farms rely on similar classes of antibiotics to fight infectious diseases. Both pathogenic and non-pathogenic bacteria evolve or exchange the ability to survive when exposed to these antibiotics. They spread into the environment through different routes, such as water sanitation systems (1), as wastewater treatment facilities do not entirely remove antibiotic resistant bacteria before releasing water into the environment. Another common route is through the application of manure to fields with cultivated crops (2), where antibiotic resistant bacteria can readily develop on the plants (3). The uptake of these resistant bacteria can then happen through the food chain, when humans later consume these plants (4) or the contaminated flesh of animals and fish harbouring resistant bacteria (5). As bacteria can easily reach water reserves, water distribution infrastructure is also a potential route for the spread of these germs (6). Even wildlife, insects and other bugs are potential carriers of antimicrobial resistance (7). Tourism, migrations and food imports (8) are nevertheless reported as the fastest way of spreading resistant strains of bacteria across borders. At the healthcare facilities level, resistant bacteria can spread by contact between patients or with healthcare staff, or through contaminated surfaces and medical devices. *Reproduced with permission from bioMérieux (modified)*

Growing human and animal populations, international travel and trade, as well as contact with wildlife all contribute to spreading AMR and making it a global health concern. Beyond international collaboration, a One Health perspective is urgently needed as AMR involves a dynamic and complex web of interactions; there are many paths by which drug residues and resistant bacteria can disseminate between humans, animals and the environment (Fig. 2).

The highest concentrations of antibiotics and resistant bacteria have been recorded in effluents released from hospitals and drug manufacturing sites in developing countries [55–57]. In certain cases, drinking water can also play a role in dissemination, as demonstrated by CRE detected in 9 out of 19 samples of the New Delhi chlorinated water supply [58].

The food chain also has a role in spreading resistance. Resistance in the food-borne bacteria *Salmonella* and *Campylobacter* is clearly linked to antibiotic use in food animals, and food-borne diseases caused by such resistant bacteria are well documented in people [59, 60]. In a One Health perspective, food-borne *E. coli* is even more worrying as it is frequently found in retail meat and is often associated to critical ESBLs [61]. In the United States for instance, a 2013 survey revealed a 65 % prevalence of *E. coli* in retail chicken products. Some of these were extra-intestinal *E. coli* (ExPEC) that closely resembled *E. coli* isolates found in humans regarding phylogenetic group, serotype distribution and virulence factors. In chickens, ExPEC isolates had on average three more virulence-related genes than non-ExPEC isolates, but there were no significant differences in phenotypic resistance [62].

Whole genome sequencing has also demonstrated that strains of *E. coli* isolated from retail turkey, chicken, or pork products correspond closely with the etiologic agents of human urinary tract infections occurring in the same locale where the foods were purchased, suggesting food-borne transmission of meat-source *E. coli* to humans and subsequent disease. The transmission of pathogenic strains from humans to animals has also been documented, for example in the case of MRSA type CC398 [63].

In addition, plasmids carrying ESBL genes have been identified in Enterobacteriaceae of both human and animal origins. For instance, some non-ST131 *E. coli* from cattle were shown to harbor plasmids carrying the human-associated CTX-M-15 ESBL genes. And IncI1/ST3 plasmids found in the microbiota of several animal species have a role in the spread of CTX-M-1-types of ESBL in *E. coli* [64].

Both Gram-positive and Gram-negative pathogenic bacteria of human origin have been reported in animals. The same holds true for a large variety of clinically relevant resistance genes, including VIM, NDM and OXA-48 like carbapenemases, which have already been detected in pigs, dogs and several wildlife species [65]. Insects may also play a role in these genetic transfers [66], but the exact transfer rates and transmission routes are still unknown. Hence, the relative contributions of the environment, livestock and humans to AMR are still debated among experts [67].

### The way forward for efficient surveillance

Obtaining a global picture for AMR is difficult due to a lack of data and standardised surveillance methodologies. Recent WHO reports on AMR have highlighted major gaps in this area and call for closer collaboration between surveillance networks at national and international levels [18].

Countries that have adopted strategies against AMR have all included the establishment of national surveillance systems in their policies. Yet, despite WHO’s call to apply surveillance to all sectors using a One-Health approach, few countries now combine human, animal, food and environmental data in their reporting. In this regard, Denmark, Sweden and Norway have led the way, demonstrating how joint responsibility-taking by health, agriculture and environment authorities can result in robust surveillance systems. Recently, several other countries have followed suit. For instance Argentina, South Africa and the Gulf Cooperation Council have announced their intention to embrace a One Health approach against AMR. The Australian Ministries of Health and of Agriculture have issued a common strategy and have expanded surveillance through a One Health approach (with a list of monitored species now longer than the list recommended by WHO). Additionally, the US national action plan against AMR also calls for enhanced efforts, including better surveillance, in both human and animal sectors.

At country scale, there remains scope to improve the convergence of existing surveillance systems. In the United States, for instance, several monitoring networks (such as the National Healthcare Safety Network, the National Antimicrobial Resistance Monitoring System, and the Emerging Infections Program) focus on different settings or different pathogens; aggregating their data provides useful insights for enhancing the management of infectious diseases as a whole.

Surveillance gaps include the fact that many surveillance systems focus on hospitalised patients, leaving community settings under-represented. For use data, the opposite is true; for many countries, there is more data on use in the community then in hospitals. But in many cases, data on antibiotic consumption is not collected with corresponding information on clinical indications; as a result, characterising antibiotic misuse often remains challenging. The burden of disease associated with AMR is also poorly documented; surveillance systems should ideally track the clinical outcomes related to antibiotic resistance.

## Sanitation, hygiene and infection prevention measures

The first among infection prevention and control (IPC) measures to contain AMR spread is hand hygiene. Improving hand hygiene is an essential approach to combat AMR. The WHO’s global “Clean Care is Safer Care” campaign, launched in 2005, has been an opportunity for many countries to learn how to adapt a global campaign to national needs, in order to achieve local buy-in [68, 69]. It relies on a multi-modal strategy for implementation, with many practical tools, illustrations and straightforward, catchy phrases. The campaign empowers countries to participate through pledges by their health ministries, and has a self-assessment framework that could serve as a blueprint for a similar tool that would assess -- and enhance the visibility of -- efforts toward antimicrobial stewardship in hospitals. After 10 years of operation and deployment through 179 countries, the “Clean Care is Safer Care” campaign has achieved a 50 % reduction in hospital-acquired infections through improved hand hygiene, and has contributed to saving an estimated 7 or 8 million lives per year [70].

Australia joined WHO Clean Care is Safer Care in 2006. More than 850 health facilities participate in the country’s National Hand Hygiene Initiative, which has played a major role in the decline of MRSA cases in hospitals [71]. This is probably the world’s largest hand hygiene education and compliance monitoring programme, and includes a comprehensive set of tools – including apps for mobile devices – to ensure data collection and compliance.

In India, vaccination and hygiene-related policies have made impressive progress, with full immunization coverage of children now approaching the 90 % mark, and the country pledged to become “open defecation free” by 2019, with plans to build 120,000 toilets in rural India by October 2019, at a projected cost of US$31 billion.

The Gulf Corporation Countries have also adopted strategies to enhance IPC as early as 2009. The countries of the region adopted both a robust infection control manual and a surveillance manual for healthcare-associated infections (HAIs), to be updated in 2015.

### Infection control of critical Gram-negative bacteria

With the rise of pan-resistant Gram-negative bacteria, IPC measures are all the more vital for controlling outbreaks. There is no single strategy to fit all situations, but a few important lessons have been learned in this field.

In Israel, an outbreak of multidrug-resistant *A. baumannii* was kept in check by accurately and rapidly identifying the origin of the outbreak, which in this case was multiple (there were several concurrent outbreaks) [72]. As transmission routes are rarely uniform, the mapping of the “transmission opportunities” proved to be a good way of pinpointing high-risk patient groups.

Patient screening is a powerful tool for identifying transmission routes and targeting interventions. Active surveillance has often demonstrated a significant impact on reducing CP-CRE incidences, but remains a subject of ongoing debate [73]. While planning for screening procedures, it is also important to consider subsequent costs to reduce transmission, such as isolation or cohorting of colonised patients.

Deciding which parts of the body need to be screened is not always straightforward. Screening for *Acinetobacter* species may require skin, pharynx, tracheal, or perianal samples. Detection of *Pseudomonas* species may prove most complicated, as they do not selectively colonise any particular part of the body [73].

Basic IPC measures – including hand hygiene, proper use of personal protective equipment (PPE), proper cleaning and disinfection of the environment and medical equipment, and patient and visitor crowd control – are always required, but may be difficult to implement in many healthcare settings around the world. Use of antiseptic baths or wipes have been reported to have a significant impact on reducing *Acinetobacter* incidence, and environmental hygiene is an important adjunct measure that requires particular attention with pan-resistant Gram-negative bacteria [74].

France has adopted a set of guidelines for identifying CRE in clinical specimens and fecal screening. Since 2013, a set of guidelines to deal with critical MDR organisms as soon as they are detected is also being enforced (Table 2). These guidelines led to contain the spread of MRSA, VRE and carbapenemase-producing enterobacteriacae in a large set of French public hospitals [75].

**Table 2:** Summary of the French guidelines to deal with critical MDR organisms

Main steps	Main causes of failure observed
Isolating the patient, at best cohorting and dedicated staff	– Delayed measures (e.g., patients admitted over the week-end or medical staff not reacting quickly)
	– Lack of dedicated healthcare workers to implement isolation or cohorting
	– Missing readmission /admission screening of a patient known to carry a MDR organism
	– Missing information on a previous stay of the patient in another hospital, particularly in a foreign country
Alerting hospital management	– Mistakes in the hospital management system
	– Loose relationship between the infection control team and hospital management
Stopping transfers of patients to other hospitals	– Continuation of patient transfers to other hospitals
Screening any people who may have been in contact with the patient	– Uncompleted list of contacts
	– Not sampling identified contact patients
	– Missing admission of a patient transferred from a ward or hospital where outbreak is ongoing
	– Inadequate lab facilities
Reinforcing hand hygiene	– Poor hand hygiene at baseline
	– Insufficient input of infection control team
Identifying antibiotics that could be used in case of critically-resistant infections	– Delayed identification by the laboratory

## Use of antimicrobial medicines in human and animal health

Prudent use of existing antibiotics must be a priority, because their consumption is one of the main drivers of AMR [76]. Bans or restriction policies have proved effective in curbing resistance in some settings [77, 78], and have achieved impressive results in the veterinary sector [79]. Persuasive approaches may also be highly successful, if well-designed and implemented [80]. Another advantage of implementing stewardship programs is the resulting reduction in the various side effects of antibiotics [81]. One of their most common adverse events is the difficult-to-treat – and sometimes fatal – diarrhoea caused by *Clostridium difficile* infection, a condition likely to remain of high concern [82].

Despite strong evidence about the cost-effectiveness of stewardship interventions, the vast majority of AMR-related resources are currently allocated to the development of new antibiotics. This may not be the best strategy, because if we develop new agents and do small interventions in all the other areas, history is likely to repeat itself; any new drug will lose its effectiveness as soon as resistance develops in bacterial strains [83]. The Center for Disease Dynamics, Economics & Policy (CDDEP) recently estimated that investing US$ 50 million in an antibiotic stewardship programme can “buy” one full year for the billion-dollar research and development programmes in the United States that are trying to bring new antibiotics to the market [84].

In hospitals, prophylactic prescriptions of antibiotics should be the first target of stewardship interventions. Although this practice is necessary for certain perioperative and surgical procedures, treatment should not extend beyond 24 h (most frequently, a single dose is sufficient). Yet, longer regimens continue to be used in many healthcare settings around the world [85]. Some facilities have their own antibiotic prescription guidelines against which to verify compliance, but most hospitals still lack such a tool [86].

An analysis of a subset of antibiotic use data collected for the Point Prevalence Survey performed by the CDC in 2013 suggests that there are two further low hanging fruits for inpatient stewardship interventions. First, the treatment of urinary tract infections because nearly 40 % of such antimicrobial prescriptions were inappropriate, and second, vancomycin use, because in many cases treatment was continued for more than three days without evidence of an infection caused by a resistant Gram-positive organism [87].

### Antimicrobial stewardship in animal health

Denmark implemented stewardship programs in the veterinary sector as early as 1998. As a result, the amount of antimicrobials consumed per kilogram of pig meat produced was reduced by 50 % from 1994 to 2013 [88, 89]. Other successful examples of antimicrobial conservation in animal health are depicted in Table 3.

**Table 3:** Successful examples of antimicrobial stewardship in animal health

Country	Main measures implemented	Observed effects
Australia	Fluoroquinolones (FQ) not approved for livestock use	Levels of FQ resistance among *Escherichia coli* in humans are among the lowest registered. FQ resistance in *Escherichia coli* is absent in food animals and foods. There are no FQ resistant strains of *Campylobacter spp.* or *Salmonella* spp. seen in food animals or domestic foods [90]. FQ resistance is absent or only at very low levels in domestically acquired *Campylobacter* or *Salmonella* infections in people.
Canada	Voluntary withdrawal of ceftiofur in ovo use.	Thirty months later, resistance levels cut by half in *Salmonella enterica* from chicken meat and humans and in retail chicken *Escherichia coli* [91].
Denmark	Ceasing antibiotic growth promotion in weaning pigs	Ten years after, the average daily weight put on by each animal was 20 % higher than before the ban, demonstrating that weight gains in livestock are achievable by other means than antibiotics [88].
Netherlands	Usage of fluoroquinolones and third and fourth generation cephalosporins reduced to a minimum	Antimicrobial consumption in animals fell 56 % from 2007 to 2012 [92].

In 2011, a sharp increase of resistance to cephalosporins was detected in bacteria colonising indigenous broilers. This observation was unexpected, because this class of antibiotics had not been used on Danish broilers for 10 years. The explanation came from the UK, where the ancestors of the Danish broilers had been raised and had received cephalosporin treatments in their youth, selecting for ESBL-producing bacteria which were then transmitted to their offspring [93]. This is another demonstration of how the usage of antimicrobials in one country can affect resistance levels in another. In fact, the very intensive exchange of livestock within and between countries has important implications for control strategies.

Stopping the use of antibiotics as growth promoters in food animals is a long awaited measure that has been implemented in only a limited number of countries to date. A sparing use of antimicrobials for prophylaxis and therapy is also crucially needed [94]. In any case, food animals should not be treated with “critically important” or “last line” antibiotics for humans (namely, glycopeptides, fluoroquinolones, 3rd generation cephalosporins, carbapenems). Regulations on this important issue are scarce or non-existing.

## New medicines, diagnostic tools, vaccines and other interventions

### Therapeutic challenges posed by pan-resistant Gram-negative bacteria

CRE can be considered as one of the plagues of the early 21st century, as they are associated with drastically increased mortality, length of stay, and cost of treatment [95]. A study on neutropenic and non-neutropenic patients documented a 63 % mortality rate (at 28 days) among patients with bacteremia caused by carbapenem-resistant strains vs 38 % among those infected by carbapenem-sensitive strains [96].

Worryingly, the mean duration of CRE carriage following hospital discharge has been estimated to 387 days (95 % CI; 312–463) [97]. And co-carriage of several other carbapenemases seems to be common, further increasing the risk of treatment failure [98].

New drugs against MDR Gram-negative bacteria should become available in the near future, but it is unlikely that any of them will prove effective against all existing carbapenemases [99]. For the time being, critical MDR infections often require the use of old drugs and combination therapies, which frequently induce larger side effects and morbidity. Unfortunately, very limited evidence exists about the efficacy of these treatments, for instance the colistin-imipenem-tigecycline combination to treat carbapenem-resistant bacteria. Some studies with limited datasets have demonstrated the superiority of combination therapies [100, 101], whereas others did not show any significant difference [102, 103]. While studies based on larger samples are urgently needed to determine the best treatment options, resistance to these last-resort classes of antibiotics is also rising in several Gram-negative bacteria [104, 105], probably due to increased consumption: for example, between 2000 and 2010, the global use of carbapenems and polymyxins increased 45 and 13 %, respectively [2].

### Alternatives to antibiotics

**Fig. 3:**
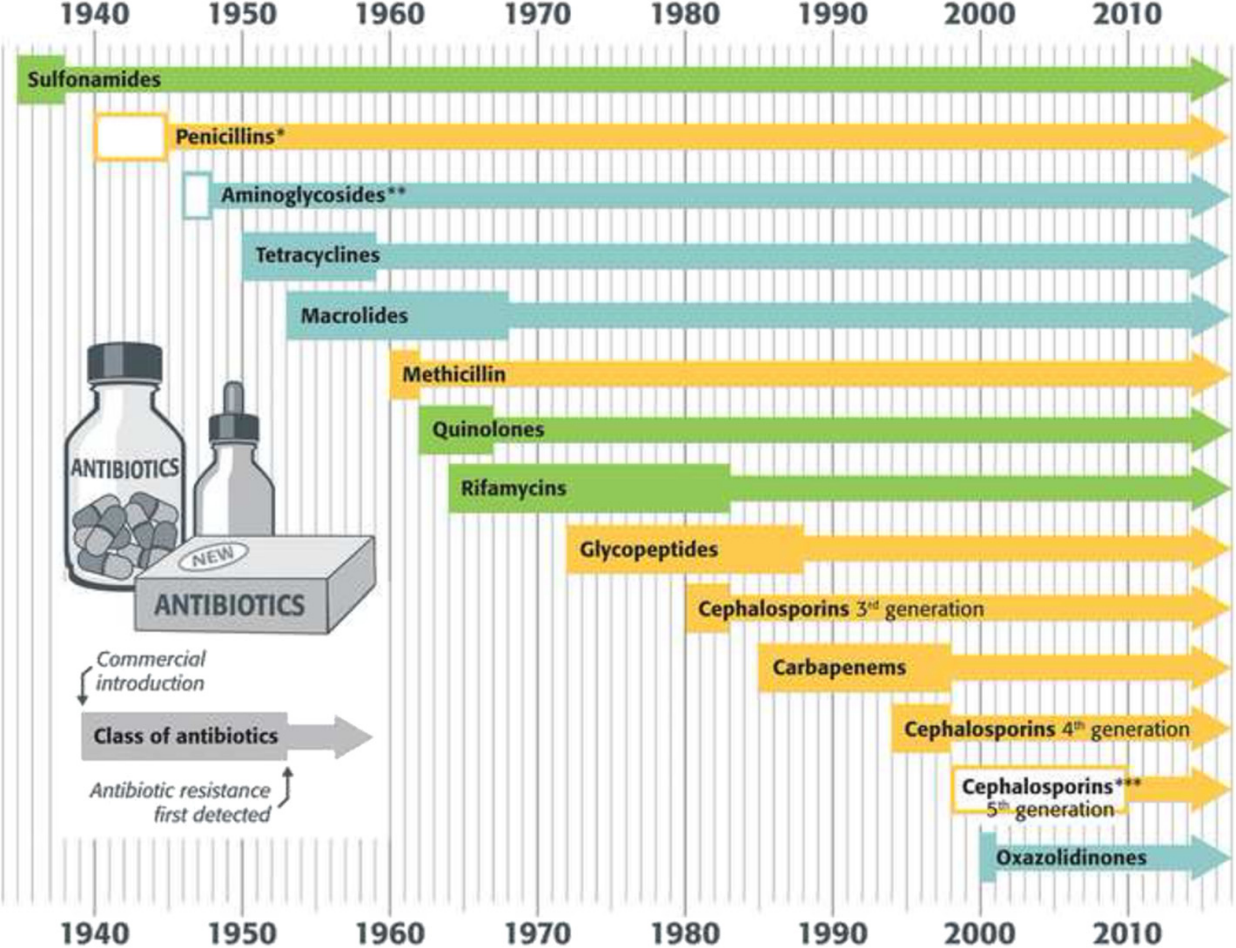
Commercialisation and first detection of resistant bacteria for some classes of antibiotics. Legend: Classes of antibiotics which disrupt the synthesis of the bacterial cell envelope are depicted in orange, those inhibiting the bacterial protein synthesis are depicted in blue, and those interfering with the metabolism of nucleic acids in bacteria are depicted in green. Empty boxes indicate molecules for which resistance has been documented prior to commercialisation. * Resistance to penicillin was observed by Fleming himself even prior to its widespread availability and commercialisation. ** Aminogylcosides: The first mutants resistant to streptomycin were reported as early as 1946, whereas its commercial introduction dates back to 1948 [113]. *** 5th generation cephalosporins: ceftaroline resistance was documented prior to its commercial introduction in 2010 [114]

Streamlining and facilitating the development of new antimicrobials is a necessary but costly option, [106] and bacterial resistance to these compounds has always emerged quickly. For many classes of antibiotics developed so far, resistance emerged on average five years after commercial release of the compounds (Fig. 3); some resistance mechanisms were already present even before the commercialisation of the agent.

Most national strategies against AMR recognise the urgent need for new antibiotics, but also for alternative solutions to circumvent the resistance problem. These may include probiotics and prebiotics, drugs targeting bacterial communication or virulence, therapies based on bacteriophages or phage enzymes, or those harnessing the power of the immune system, such as antibodies or vaccines. Vaccination may not work against all bacterial strains, but it shows great promises against some, such as uropathogenic *E. coli* (UPEC) strains responsible for the majority of urinary tract infections.

### New tools for better surveillance

Currently, culture is the key component in the detection and preliminary identification of MDR organisms. A variety of rapid diagnostic tests and selective media has been developed and used with great success in defining intra- and inter-individual colonisation and infection rates. The methods are easily available, well accepted, and affordable, but take time and effort. New diagnostic tools based on protein identification (e.g., mass spectrometry, biochemical or immunological techniques) are increasingly available at lower costs, as are tests characterising other biomolecules, including for instance lipids and sugars. These can provide further epidemiological insights – compared to simple phenotypic tests – and many of them are easy to use in routine laboratory work [107].

Gene identification, which relies on the detection of known resistance genes, is also becoming more accessible, but some of the commercially available tests are unable to discern between gene variants (for instance between oxacillinases 48, 163, and 405). False positives can also be an issue, as detected resistance genes may be present in non-pathogenic bacteria, or corresponding minimum inhibitory concentrations can in fact be very low. In addition, genes may be present but expressed poorly or not at all.

Progress has been made with genome sequencing – now available at lower costs. It delivers results within 18–24 h and, through the identification of clonal relationships, could be a powerful tool to reveal the source of an outbreak and its dissemination routes. For instance, the genetic mapping of integrons during a small outbreak of KPC-2 in Israel revealed its route of transmission and complex polyclonal evolution, involving several intermediate hosts (*E. cloacae* - > *E. coli* - > *K. pneumoniae*) [108]. Finally, in some cases, genome sequencing has also proved to be more robust than phenotypic antimicrobial sensibility testing [109]. Still, genomic epidemiology needs to be further calibrated taking into account genome-wide mutational frequencies during dissemination or growth under unfavorable conditions. In addition, the technology should be simplified and data interpretation and reporting should be automated.

## Conclusion

Lectures and discussions during the fifth World Healthcare-Associated Infections Forum highlighted some measures of progress along the most urgent priorities for action cited by participants of the Forum’s earlier editions [110, 111]. While the best strategies to curb AMR are becoming obvious, isolated country based efforts will not be sufficient and further research is direly needed in several fields. The links between antibiotic use and resistance genes that circulate in livestock, humans and the environment remain poorly understood; for instance, available data do not allow accurate quantification of the contribution of antimicrobial consumption in one sector to resistance in another. Our understanding of the ecological roles of antibiotics in nature is incomplete, in particular regarding the effects of sub-inhibitory levels in the environment; the levels of antibiotic contamination at which resistant bacteria are selected for and horizontal gene transfer is facilitated should be determined [112]. Further investigation of “cross resistance” and “co-selection” mechanisms is also warranted. Last but not least, more investments are required for enabling behaviour change.
